# Prevalence and factors associated with pre-diabetes and undiagnosed diabetes in Cambodia: cross-sectional study based on the World Health Survey Plus 2023

**DOI:** 10.1136/bmjopen-2025-102715

**Published:** 2026-01-14

**Authors:** Srean Chhim, Grace V Ku, Paul Kowal, Vannarath Te, Mony Sorithisey, Chamnab Ngor, Poppy Walton, Khin Thiri Maung, Nawi Ng, Nirmala Naidoo, Ir Por, Kerstin Klipstein-Grobusch, Chhorvann Chhea, Heng Sopheab

**Affiliations:** 1 School of Public Health, National Institute of Public Health, Phnom Penh, Cambodia; 2 Julius Global Health, Department of Global Public Health & Bioethics, Julius Center for Health Sciences and Primary Care, University Medical Center Utrecht, Utrecht University, Utrecht, Netherlands; 3 Department of Public Health, Institute of Tropical Medicine Antwerp, Antwerp, Belgium; 4 Department of Gerontology, Faculty of Medicine and Pharmacy, Vrije Universiteit Brussel, Brussels, Belgium; 5 Health Data Analytics Team, National Centre for Epidemiology and Population Health, Australian National University, Canberra, Australian Capital Territory, Australia; 6 HelpAge International, London, UK; 7 School of Public Health and Community Medicine, Institute of Medicine, Sahlgrenska Academy, University of Gothenburg, Goteborg, Sweden; 8 Department of Epidemiology and Global Health, Umea University Faculty of Medicine, Umea, Sweden; 9 Department of Data and Analytics, Division of Data, Analytics and Delivery for Impact, World Health Organization, Geneva, Switzerland; 10 Management team, National Institute of Public Health, Phonm Penh, Cambodia; 11 Division of Epidemiology and Biostatistics, School of Public Health, Faculty of Health Sciences, University of the Witwatersrand Johannesburg, Johannesburg, South Africa

**Keywords:** Epidemiology, Diabetes Mellitus, Type 2, Health Surveys

## Abstract

**Abstract:**

**Objective:**

This study aimed to determine the prevalence and factors associated with pre-diabetes and undiagnosed type 2 diabetes (UDD) in Cambodia.

**Design:**

This analysis used data from the WHO World Health Survey Plus, which was collected using a cross-sectional design with a GIS-based, three-stage sampling approach. Multiple logistic regression was used to identify key associated factors, based on a significance level of p<0.05.

**Setting:**

Data were collected from all 25 provinces in Cambodia between 12 March 2023 and 31 May 2023.

**Participants:**

4427 individuals aged 18 years or older, residing in the selected household for at least 6 months in the past year.

**Primary outcome measures:**

Pre-diabetes (Haemoglobin A1c (HbA1c) 5.7%–6.4%) and UDD (HbA1c≥6.5%), without prior knowledge of having type 2 diabetes (T2D).

**Results:**

The weighted prevalences of pre-diabetes and UDD were 26.4% (95% CI 24.0% to 29.0%) and 9.3% (95% CI 7.9% to 11.0%). Pre-diabetes prevalence was higher in urban areas compared with rural areas (adjusted OR, aOR=1.2, 95% CI 1.0 to 1.4), males (aOR=1.7, 95% CI 1.3 to 2.3), individuals aged 40–49 (aOR=1.8, 95% CI 1.4 to 2.4), individuals aged 50+ years group (aOR=2.9, 95% CI 2.3 to 3.6) compared with the 18–39 years group, overweight individuals (aOR=1.7, 95% CI 1.4 to 2.0), obese (aOR=2.1, 95% CI 1.5 to 3.0) and those with elevated total triglycerides (aOR=1.3, 95% CI 1.1 to 1.5). Similar risk factors were identified for UDD, with the addition of hypertension (aOR=1.6, 95% CI 1.3 to 2.0) and high waist circumference (aOR=2.0, 95% CI 1.5 to 2.7).

**Conclusions:**

The high prevalence of pre-diabetes and UDD in Cambodia is a pressing public health concern. Urgent and intensive interventions are needed to effectively prevent and manage T2D, particularly among urban residents, older persons and individuals with metabolic risk factors.

**STRENGTHS AND LIMITATIONS OF THIS STUDY**

- The study used a large, nationally representative sample to examine pre-diabetes and undiagnosed type 2 diabetes (T2D) in Cambodia.
- HbA1c was measured and used for determining pre-diabetes and T2D, and is considered a reliable measure of glycaemic status within populations.
- Limitations include the cross-sectional design, which prevents drawing conclusions about cause and effect between predictors and the outcome; reliance on self-reported lifestyle factors, which may be subject to recall bias; and the absence of data on family history and other significant risk factors.

## Introduction

 Type 2 diabetes (T2D) continues to be a leading disease burden and represents a significant global public health challenge.^1^ Adding to this challenge are populations with pre-diabetes that have blood sugar levels higher than normal but not high enough to be diagnosed with T2D.^2^ Specialised institutions providing guidance on T2D prevention and management like the WHO, American Diabetes Association (ADA) and International Expert Committee (IEC) define pre-diabetes inconsistently, with at least five definitions identified: (1) ADA: fasting blood glucose (FBG) 100–125 mg/dL; (2) WHO: FBG 110–125 mg/dL; (3) ADA/WHO: 2-hour post-load blood glucose (2-hour BG) 140–199 mg/dL; (4) WHO: HbA1c 5.7%–6.4% and (5) IEC: HbA1c 6.0%–6.4%.^2^

Up to 70% of individuals with pre-diabetes may progress to T2D, with the estimated prevalence increasing exponentially.^3^ The global prevalence of pre-diabetes, as measured by the ADA’s FBG criteria, was 9.1% (464 million) in 2021 and is projected to increase to 10.0% (638 million) by 2045.^4^ The prevalence of pre-diabetes tended to be higher when measured using HbA1c.^5^,^9^

Furthermore, approximately half of the global population with T2D remains unaware that they have T2D, which increases the likelihood of complications and hinders effective T2D case management.^10^

Certain risk factors for T2D, such as age, sex, genetics, ethnicity, history of gestational diabetes, delivery of large-for-gestational-age infants and menopause, are non-modifiable; however, addressing modifiable risk factors can mitigate the risk of developing pre-diabetes and T2D.^11 12^ Modifiable risk factors include unhealthy dietary habits, physical inactivity, tobacco use, excessive alcohol consumption, poor stress management and poor sleep patterns. These are also risk factors for metabolic syndrome and a constellation of intermediate risks, including elevated blood pressure and abnormal cholesterol/triglyceride levels.^11 13^

In Cambodia, the prevention, screening and management of T2D face challenges within the pluralistic healthcare system. One study assessed key components of integrated T2D care and found a low implementation score in early detection, primary care treatment, health education, self-management support, structured collaboration and care organisation.^14^ Furthermore, only 10% of adult general outpatient visits and 35% of visits among people with T2D occurred in public facilities, indicating that most visits were made to the private sector, where patients were required to pay out-of-pocket for health expenses.^12 15^

Research on pre-diabetes and undiagnosed type 2 diabetes (UDD) in Cambodia is limited. In 2023, a nationally representative non-communicable disease (NCD) risk factor survey using FBG to examine the prevalence of T2D and its associated risk factors (physical inactivity, alcohol consumption, tobacco use, sodium intake, poor diet and high blood pressure).^16^ This study estimated pre-diabetes prevalence at 4.5% but did not focus on prevalence of UDD.^16^ Another study in Cambodia assessed the proportion and risk factors of UDD but focused on individuals aged 40 years and older, excluding younger adult populations who may also be at risk.^17^

To address these gaps, this study aimed to contribute to the understanding of T2D in Cambodia by examining the prevalence and factors associated with pre-diabetes and UDD. These results provide valuable insights into the development of effective T2D prevention and management strategies in Cambodia.

### Conceptual framework

**Figure 1:**
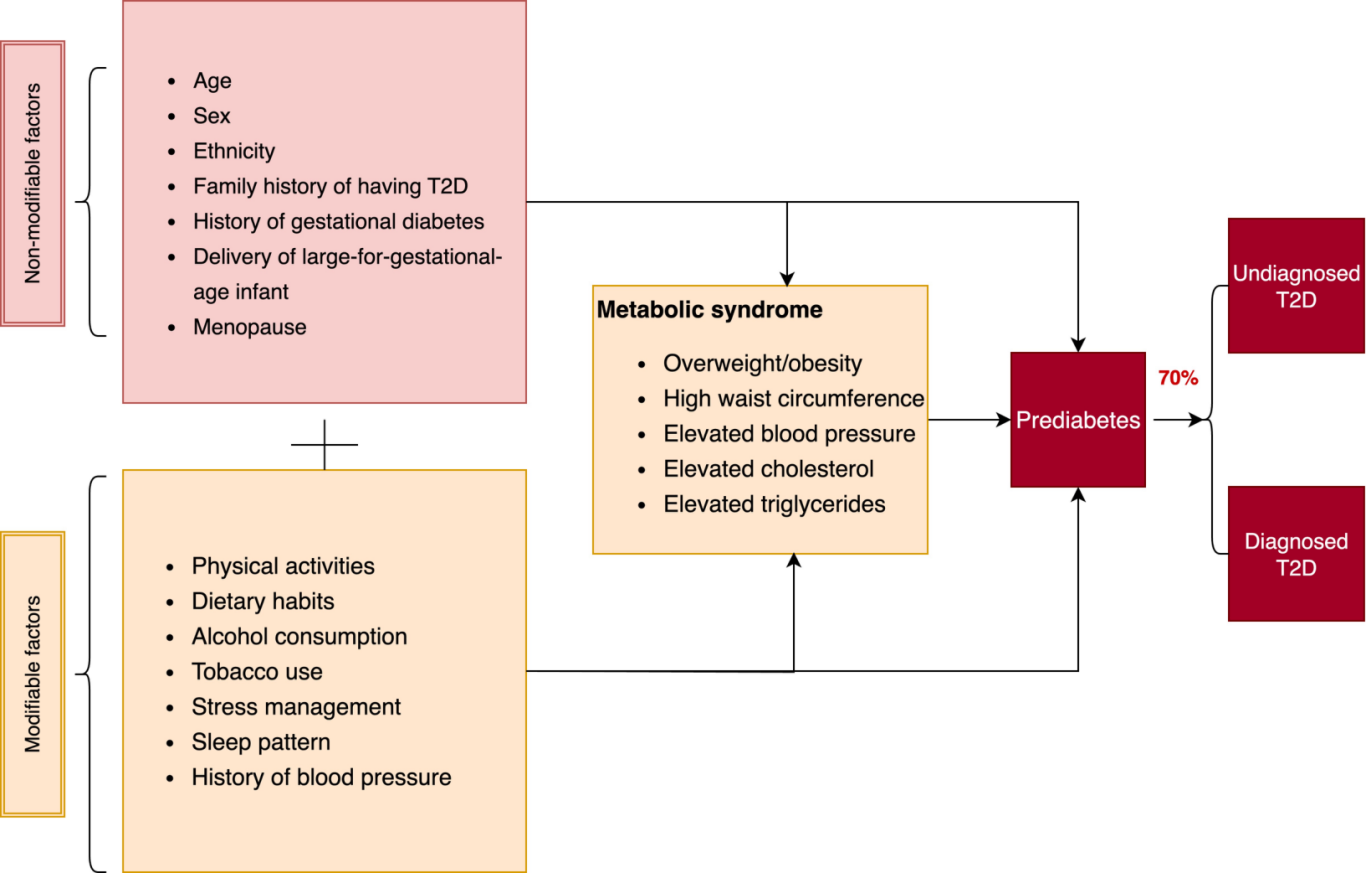
T2D, type 2 diabetes.

The prevalence and factors associated with pre-diabetes and UDD have been extensively studied in other settings, including Southeast Asia.^18^,^24^ Pre-diabetes and UDD typically have a mix of non-modifiable and modifiable risk factors (figure 1).^25^

### Conceptual framework of the potential association between risk factors and pre-diabetes and UDD

Previous studies have shown that age is an established independent factor, as human cells become resistant to insulin or the pancreas produces insufficient insulin with advancing age. Age can also be associated with reduced physical activity, leading to weight gain and an increased risk of pre-diabetes and T2D. Physical activity and other factors, including dietary habits, alcohol consumption, tobacco use, stress management and sleep habits, can be interrelated and strongly associated with metabolic syndrome before developing T2D.^26^,^28^ The analysis of pre-diabetes and UDD factors should consider a multifaceted approach that includes non-modifiable and modifiable factors. This approach can help identify high-risk individuals and inform targeted interventions for early detection and management of T2D.

## Methods

### Data sources

This nationally representative cross-sectional study used data from Cambodia’s 2023 World Health Survey Plus (WHS+).^29^ The WHS+is a WHO-led global tool tracking progress towards health-related goals in low-income and middle-income countries.^29^ The National Institute of Public Health implemented the survey in Cambodia, with technical and logistic support from WHO, HelpAge International, the University of Gothenburg (Sweden), the Australian National University (Australia) and the University of Oregon (USA). The study involved interviews with a nationally representative sample of adults aged ≥18 years from selected households nationwide. The original protocol is available in online supplemental file 1.

### Sampling and recruitment process

A three-stage sampling process was used for the WHS+. First, 276 of the 14 568 villages were randomly chosen across Cambodia, with a selection probability proportional to the village size. Second, we selected households. High-resolution satellite images and Geographic Information System (GIS) software were used to map all buildings within each selected village. From this, 44 buildings were randomly selected based on the pretesting data, indicating that approximately 50% would contain eligible households. Households were eligible if they had at least one permanent resident aged 18 years or older who had lived there for at least 6 months in the last 12 months (n=6154). Within each household, one eligible adult was randomly selected for an individual interview, and the household head completed a separate household questionnaire. Of 6154 eligible households, 5271 completed the survey, yielding a response rate of 85.6%. For the prevalence calculation, 844 participants were excluded due to unavailable HbA1c results, resulting in a final analysed sample of 4427 (online supplemental figure 1).

### Data collection

Data were collected between 12 March 2023 and 31 May 2023, by 14 teams, each consisting of a leader, interviewers and staff responsible for completing biomarker and performance tests. All team members underwent a 10-day training before data collection began.

HbA1c was tested using the A1CNow point-of-care testing device, which requires approximately 5 µL of blood sample and provides results within 5 min.^30^ The device features a built-in quality control function to ensure reliable results. It automatically detects issues such as insufficient or excessive blood volume, abnormal haemoglobin density and extreme temperatures.^30^ This device is designed for research purposes or as a diagnostic tool when laboratory-based HbA1c is not available.^31^ A systematic review and meta-analysis analysed biases using A1cNow, reporting a low average bias ranging from –0.70% (95% CI –0.82% to 0.58%) to +0.67% (95% CI 0.52% to 0.82%), compared with laboratory-based testing.^32 33^

During the survey, lipids and random blood glucose levels were also measured using a CardioChek PA Analyser (PTS Diagnostics Headquarters, Whitestown, Indiana, USA)^31^ and blood pressure using an Automatic Blood Pressure Monitor HEM-7120 (OMRON Healthcare, Kyoto, Japan).^34^

### Measures

The primary outcomes were pre-diabetes and UDD.

- Pre-diabetes was defined using WHO’s HbA1c criteria (5.7%–6.4%). For the risk factor analysis, the comparison group included individuals without T2D or HbA1c<5.7%, while those diagnosed or undiagnosed with T2D were excluded.
- UDD was defined as an HbA1c level >6.5% without prior diagnosis by a healthcare professional. The same comparison group and exclusion criteria used for pre-diabetes were used for risk factor analysis.

Pre-diabetes was defined using WHO’s HbA1c criteria (5.7%–6.4%). For the risk factor analysis, the comparison group included individuals without T2D or HbA1c<5.7%, while those diagnosed or undiagnosed with T2D were excluded.

UDD was defined as an HbA1c level >6.5% without prior diagnosis by a healthcare professional. The same comparison group and exclusion criteria used for pre-diabetes were used for risk factor analysis.

The explanatory variables included place of residence (urban or rural), sex (male or female), age (18–29, 30–39, 40–49, >50 years), level of education (none, primary, secondary, high school or higher) and wealth quintile, using principal component analysis (ranging from poorest to wealthiest). The urban and rural classifications followed the criteria set by the National Institute of Statistics. Additional factors were considered, such as obesity, physical activity, tobacco use, waist circumference, fruit and vegetable consumption, high blood pressure, total cholesterol, triglycerides and alcohol consumption. Additional details on the measurement and definition of these variables are presented in table 1.

**Table 1:** Categories and measurement of metabolic risk factors

Variable	Category and measurement
Obesity	Underweight (BMI<18.5), normal weight (18.5≤BMI≤25), overweight (25≥BMI<30), obese (BMI≥30)^58^
Physical activities	Weekly physical activity was assessed using Global Physical Activity Questionnaire and converted to Metabolic Equivalents of Task (MET) minutes.^6070^,^72^ Participants self-reported the frequency and duration of moderate-intensity and vigorous-intensity physical activity at work, non-work and commuting. MET values were assigned (four for moderate and eight for vigorous), and the total weekly MET minutes were calculated.^6070^,^72^ Participants were then classified into two groups: <600 MET-min and ≥600 (recommended).^60 70 72^
Tobacco use	Participants were classified as current tobacco users (current smoking/smokeless use daily or less than daily), former users (previous smoking/smokeless use daily or less than daily, but not currently) and non-users. Electronic cigarette smokers were also included in this study.
Waist circumference	Normal (male: ≤102 cm, female: ≤88 cm), High (male: >102 cm, female: >88 cm)
Fruit and vegetable consumption	Fruit and vegetable consumption was classified as meeting WHO recommendations (≥5 servings per day or approximately 400 g of fruit and vegetables combined) or not (<5 servings per day).^61^
High blood pressure	High blood pressure is classified as ‘never’, ‘ever’ and ‘uncontrolled’. Uncontrolled blood pressure is defined as an average systolic ≥140 mm Hg or diastolic ≥90 mm Hg from the last two of three measurements.^62^ ’Ever‘ refers to individuals diagnosed with hypertension but not classified as uncontrolled during the survey. ‘Never‘ denotes those not diagnosed with hypertension by health professionals or detected in the survey.
Total cholesterol level	Elevated (≥240 mg/dL)^63^
Triglyceride levels	Elevated (≥150 mg/dL)^73^
Alcohol consumption	Low risk, high risk (assessed using Alcohol Use Disorders Identification Test- Consuption (AUDIT-C))^74^

BMI, body mass index.

### Analysis

Both descriptive and inferential statistical methods were used for the analysis. Weighted results were used for univariate and bivariate analyses, whereas unweighted results were used for multivariate analyses. Records with missing HbA1c results were excluded, but missing values for explanatory variables were imputed to preserve the sample size. After removing 844 records with missing outcome values, 680 data records remained missing across several independent variables: total cholesterol (n=288), total triglycerides (n=255), insurance entitlement (n=66), body mass index (BMI) category (n=44), waist circumference (n=19), MET (n=5) and hypertension status (n=3).

To address this issue, we performed multiple imputation based on Multiple Imputation by Chained Equations package in R. The imputation model included all independent variables and T2D status. Categorical variables were imputed using logistic regression, while continuous variables were imputed using predictive mean matching. The procedure generated 10 complete datasets over 100 iterations, and all imputed variables successfully converged. The results from unimputed data using case-available analysis, where missing values were recorded as missing and retained in the model, are available in online supplemental table 2.

The descriptive analysis employed sampling weights to match Cambodia’s age-sex population and urban/rural distribution, using proportions and frequencies for categorical variables and means and SD for continuous variables. The bivariable analysis used χ² or Fisher’s exact tests for categorical variables related to pre-diabetes and UDD. Variables with a p value of 0.2 or lower were included in the multivariable analysis, using generalised linear models with binomial specification. Multicollinearity was checked using the variance inflation factor, with a cut-off value of 5. An interaction term was identified between sex and age in pre-diabetes and UDD analysis and was included in the model. Analysis and reporting were conducted using the gt_summary and MASS packages in R with a significance threshold of p<0.05. R codes are provided as online supplemental file 2.

### Patients and public involvement

None of the participants was involved in developing the research questions, outcome measures, study design or recruitment.

## Results

### Participant characteristics

#### Sociodemographic characteristics

As shown in table 2, this study included 4427 participants, most of whom were female, with a weighted proportion of 52.2%, and lived in rural areas (59.1%). The weighted average age was 41.0 years, with the most significant proportion in the 18–29 age group (29.7%). Most participants were married or living together (80.3%) and had low educational attainment, with 67.9% having completed primary school or less.

**Table 2:** Sociodemographic characteristics of participants

Characteristic	Unweighted	Weighted	
	n=4427	N=4427	95% CI
Type of community			
Rural	2750 (62.1)	59.1	57 to 62
Urban	1677 (37.9)	40.9	38 to 43
Sex of participant			
Male	1336 (30.2)	47.8	45 to 50
Female	3091 (69.8)	52.2	50 to 55
Age in years (mean (SD))	47.3 (14.9)	41.0	40 to 42
Age group (years)			
18–29	570 (12.9)	29.7	27 to 32
30–39	909 (20.5)	23.6	21 to 26
40–49	922 (20.8)	17.7	16 to 20
50+	2026 (45.8)	29.0	27 to 31
Marital status			
Married or living together	3452 (78.0)	80.3	78 to 82
Never married	242 (5.5)	11.3	9.6 to 13
Separated /divorced/widowed	733 (16.6)	8.5	7.3 to 9.8
Socioeconomic class			
Poorest	873 (19.7)	13.8	12 to 16
Poor	886 (20.0)	16.6	15 to 18
Middle	897 (20.3)	20.8	19 to 23
Rich	891 (20.1)	21.0	19 to 23
Richest	880 (19.9)	27.9	26 to 30
Education level			
Never attended school	979 (22.1)	14.6	13 to 16
Less than primary school	1545 (34.9)	27.1	25 to 29
Primary school completed	1000 (22.6)	26.2	24 to 29
Secondary school completed	537 (12.1)	18.0	16 to 20
High school completed	245 (5.5)	9.2	7.8 to 11
College/preuniversity/ university completed	121 (2.7)	4.9	3.8 to 6.2

### Modifiable risk characteristics

One-third of the participants were classified as physically inactive with a weighted proportion of 35.2%, and 25.0% were current or former tobacco users (table 3). About a quarter (24.4%) of the participants were at high risk of alcohol use disorders. Approximately 56.4% of the participants consumed fewer than five servings of fruits and vegetables daily, with an average of 2.0 servings of fruits and 2.4 of vegetables. The average BMI was 23.7; 27.7% of the participants were overweight, 6.1% were obese and 10.9% had a high waist circumference. Elevated triglyceride and total cholesterol levels were found in 47.3% and 12.8% of the participants, respectively. Hypertension was present in 29.6% of participants, whereas 14.2% had previously been diagnosed with hypertension.

**Table 3:** Modifiable risk characteristics of participants

Characteristic	Unweighted	Weighted	
	n=4427	N=4427	95% CI
MET category			
Active (≥600)	3027 (68.5)	64.8	62 to 67
Inactive (<600)	1395 (31.5)	35.2	33 to 38
Fruit consumption per typical day (mean (SD), mean (95% CI))	2.1 (1.9)	2.0	1.9 to 2.1
Vegetable consumption per typical day (mean (SD), mean (95% CI))	2.5 (1.7)	2.5	2.4 to 2.6
Fruit and vegetable consumption			
<5	2518 (56.9)	56.4	54 to 59
≥5 (adequate)	1909 (43.1)	43.6	41 to 46
Tobacco use			
Never used	3254 (73.5)	75.0	73 to 77
Current or former user	1173 (26.5)	25.0	23 to 27
Alcohol disorder category			
Low risk	3533 (79.8)	75.6	73 to 78
High risk	894 (20.2)	24.4	22 to 27
BMI (mean (SD), mean (95% CI))	23.9 (4.1)	23.7	23 to 24
BMI category			
Underweight (<18.5)	324 (7.4)	9.1	7.7 to 11
Normal (18.5–24.9)	2472 (56.4)	57.1	55 to 60
Overweight (25.0–29.9)	1278 (29.1)	27.7	25 to 30
Obese (≥30.0)	311 (7.1)	6.1	4.9 to 7.4
Waist circumference category (cm)			
High	680 (15.4)	10.9	9.5 to 13
Normal	3729 (84.6)	89.1	87 to 91
Total cholesterol			
Normal	3529 (84.5)	87.2	85 to 89
Elevated (≥240 mg/dL)	648 (15.5)	12.8	11 to 15
Total triglycerides			
Normal	2096 (49.8)	52.7	50 to 55
Elevated (≥150 mg/dL)	2111 (50.2)	47.3	45 to 50
Having hypertension	1621 (36.6)	29.6	27 to 32

BMI, body mass index; MET, Metabolic Equivalents of Task.

### Prevalence of pre-diabetes and undiagnosed diabetes

Of the 4427 individuals included in the prevalence calculation, the weighted prevalence of pre-diabetes and UDD was 26.4% (95% CI 24.0% to 29.0%) and 9.3% (95% CI 7.9% to 11.0%), respectively, with a prevalence of diagnosed T2D of 6.7% (95% CI 5.5% to 8.0%).

### Bivariate analysis of factors associated with pre-diabetes and UDD

Bivariate analysis identified potential variables associated with pre-diabetes and/or UDD (online supplemental table 1). A higher prevalence of pre-diabetes and UDD is associated with older age, living in urban areas, higher BMI, higher waist circumference, hypertension, elevated triglyceride levels and being male. Elevated total cholesterol level was associated with a higher prevalence of UDD only.

### Multivariable logistic regression of factors associated with pre-diabetes and UDD

Table 4 presents variables independently associated with a high prevalence of pre-diabetes and/or UDD in multivariable analysis.

**Table 4:** Risk factors associated with pre-diabetes and UDD

Characteristic	Pre-diabetes			Undiagnosed T2D		
	OR	**95% CI**	P value	OR	**95% CI**	P value
Type of community						
Rural (Ref.)	—	—		—	—	
Urban	1.2	1.0 to 1.4	**0.018**	1.4	1.1 to 1.7	**0.002**
Sex of participant						
Female (Ref.)	—	—		—	—	
Male	1.7	1.3 to 2.3	**<0.001**	1.4	0.8 to 2.3	0.229
Age group (years)						
18–39 (Ref.)	—	—		—	—	
40–49	1.8	1.4 to 2.4	**<0.001**	2.2	1.5 to 3.2	**<0.001**
50+	2.9	2.3 to 3.6	**<0.001**	3.5	2.5 to 4.9	**<0.001**
MET category						
Active (≥600) (Ref.)	—	—		—	—	
Inactive (<600)	1.0	0.9 to 1.2	0.570	1.1	0.9 to 1.4	0.532
Tobacco users						
Never (Ref.)	—	—		—	—	
Former and current users	1.1	0.9 to 1.3	0.309	1.0	0.8 to 1.3	0.916
Alcohol disorder category						
Low risk (Ref.)	—	—		—	—	
High risk	0.9	0.8 to 1.1	0.335	1.2	0.9 to 1.5	0.302
BMI category						
Normal (18.5–24.9) (Ref.)	—	—		—	—	
Underweight (<18.5)	1.1	0.9 to 1.5	0.418	0.6	0.3 to 1.0	**0.050**
Overweight (25.0–29.9)	1.7	1.4 to 2.0	**<0.001**	1.5	1.2 to 1.9	**0.002**
Obese (≥30.0)	2.1	1.5 to 3.0	**<0.001**	2.6	1.7 to 3.9	**<0.001**
Waist circumference category						
Normal (Ref.)	—	—		—	—	
High	1.2	1.0 to 1.6	0.107	2.0	1.5 to 2.7	**<0.001**
Total cholesterol						
Normal (Ref.)	—	—		—	—	
Elevated (≥240 mg/dL)	1.2	0.9 to 1.4	0.198	1.3	1 to 1.7	0.111
Total triglycerides						
Normal (Ref.)	—	—		—	—	
Elevated (≥150 mg/dL)	1.3	1.1 to 1.5	**0.005**	1.7	1.3 to 2.1	**<0.001**
Having hypertension						
No (Ref.)	—	—		—	—	
Yes	1.1	0.9 to 1.2	0.533	1.6	1.3 to 2.0	**<0.001**
Sex of participant×age group (years)						
Male×40–49 (Ref=female×40–49)	0.6	0.4 to 0.9	**0.020**	0.5	0.2 to 1.0	0.052
Male×50+ (Ref=female×50+)	0.6	0.4 to 0.8	**0.001**	0.7	0.4 to 1.3	0.280

A Bold P-value indicates significance at the 0.05 level

BMI, body mass index; MET, Metabolic Equivalent of Tasks; Ref, Reference; T2D, type 2 diabetes; UDD, undiagnosed diabetes.

### Factors independently associated with pre-diabetes

Higher odds of pre-diabetes were observed in individuals residing in urban areas (adjusted OR, aOR=1.2, 95% CI 1.0 to 1.4), male (aOR=1.7, 95% CI 1.3 to 2.3), individuals aged 40–49 (aOR=1.8, 95% CI 1.4 to 2.4), individuals aged 50+years group (aOR=2.9, 95% CI 2.3 to 3.6) compared with the 18–39 years group. Higher odds were also observed in individuals categorised as overweight (aOR=1.7, 95% CI 1.4 to 2.0), obese (aOR=2.1, 95% CI 1.5 to 3.0) and those with elevated total triglycerides (aOR=1.3, 95% CI 1.1 to 1.5). Although males are generally at a higher risk, an interaction between sex and age was identified. The odds of having pre-diabetes in males aged 40–49 were lower (aOR=0.6, 95% CI 0.4 to 0.9), and in males aged 50 and above (aOR=0.6, 95% CI 0.4 to 0.8), compared with females in the same age range.

### Factors independently associated with UDD

Higher odds of UDD were observed in urban residents (aOR=1.4, 95% CI 1.1 to 1.7) and individuals aged 40–39 (aOR=2.2, 95% CI 1.5 to 3.2), individuals aged 50+ (aOR=3.5, 95% CI 2.5 to 4.9). UDD was also more likely in individuals who were overweight (aOR=1.5, 95% CI 1.2 to 1.9), obese (aOR=2.6, 95% CI 1.7 to 3.9), had high waist circumference (aOR=2.0, 95% CI 1.5 to 2.7), elevated total triglyceride levels (aOR=1.7, 95% CI 1.3 to 2.1) and hypertension (aOR=1.6, 95% CI 1.3 to 2.0).

## Discussion

Our investigation showed a high prevalence of pre-diabetes and UDD among Cambodian adults aged 18 years or older, at 26.4% and 9.3%, respectively. A higher prevalence of pre-diabetes was associated with urban residency, an interaction between older age and sex, being overweight or obese and elevated triglycerides. The same factors were associated with undiagnosed diabetes (UDD), with hypertension as an additional risk factor.

Comparing our prevalence to the global prevalence presents challenges owing to variations in diagnostic tools and cut-off criteria. While there is no consensus on pre-diabetes screening strategies, studies using HbA1c have reported a higher prevalence than those using FBG, but a lower prevalence than OGTT, which is often considered the superior method.^5^,^79 35^ Our pre-diabetes prevalence was approximately threefold the globally estimated 10% based on FBG.^3^ Regional comparisons also proved challenging because of the different age ranges of the study population. However, when considering only HbA1c studies, our pre-diabetes prevalence was in the range of prevalences from regional studies, including lower than the reported prevalence of 34.6% in Vietnam (2015, ≥20 years),^8^ but higher than the reported prevalence of 14.2% in Malaysia (2006–2012, ≥35 years),^38^ and 21.7% in China (2010, ≥18 years).^39^ Our pre-diabetes prevalence was also in the range of some European studies, including 24.7% in Swiss adults (25–41 years)^5^ and approximately 30% in Spanish adults (≥18 years).^10^ In the Cambodian context, the WHS+HbA1c-based pre-diabetes prevalence is approximately five times that of impaired fasting glycaemia, as defined by plasma venous values of 110 mg/dL and 126 mg/dL in the 2023 Cambodian NCD risk factor surveillance survey, known as STEPS 2023.^16^ These results revealed a significant prevalence of potential future T2D in Cambodia, highlighting the urgent need for public health measures to address this concern.

The observed 9.3% prevalence of UDD consisted of approximately 60% of all T2D cases in the WHS+study (detected by HbA1c or self-reported). This proportion is above the 55.2% previously reported for Cambodia by Te *et al*^17^ and markedly higher than previously published estimates for the Southeast Asian region at 51.3%,^40^ suggesting potential gaps in screening, healthcare access and disease awareness.

The risk factors analysed for pre-diabetes and UDD used a single control group of normoglycaemic individuals without diabetes or pre-diabetes. Advanced age was a significant predictor for both pre-diabetes and UDD, with risk escalating substantially with age. Notably, the prevalence of pre-diabetes and UDD among individuals aged 30–39 was 29.7% and 5.7%, respectively, and these rates continued to rise progressively in those aged 40–49 and 50+. This aligns with prior research identifying age as a non-modifiable risk factor for T2D.^718 19 21 27 41^,^45^ Biological mechanisms include increasing insulin resistance and pancreatic beta-cell dysfunction over time, which contribute to elevated blood sugar levels and T2D development.^46^ Interestingly, for both pre-diabetes and UDD, an interaction term between age and sex was observed. Males exhibited a higher prevalence of pre-diabetes and UDD earlier, whereas females surpassed males in terms of prevalence at 40 years and older, possibly owing to their inherent biological advantages. Older females are likely to encounter more significant behavioural risks than males.^46^ These results indicate that interventions should be tailored based on age and sex to maximise effectiveness.

Urban residence was associated with higher odds of developing both pre-diabetes and UDD, likely because of urban lifestyle and environmental factors. A prior study in Cambodia also reported a higher prevalence of overweight and obesity in urban populations.^47^ To the best of our knowledge, in the Cambodian context, urban settings often promote dietary shifts towards processed foods, increased sedentary behaviour or reduced physical activity, all of which contribute to metabolic risk.

This study also observed pre-diabetes and UDD prevalence to be strongly associated with overweight and obesity, elevated triglyceride levels and elevated blood pressure. This finding is consistent with previous findings that obesity and high waist circumference are independent risk factors associated with T2D.^4648^,^51^ Individuals who are overweight or obese tend to accumulate more visceral fat, impairing insulin sensitivity and increasing the risk of T2D. Similarly, elevated triglyceride and blood pressure levels are associated with insulin resistance, a key factor in T2D development.^46 48 49 52^ In the final model, high waist circumference was not associated with pre-diabetes but remained an associated factor for UDD. However, when BMI was removed from the model, waist circumference became a significant predictor for pre-diabetes. This indicates that BMI and waist circumference are highly correlated, suggesting that either measure can be used to predict pre-diabetes and UDD. This is a notable finding that can be utilised in settings where there are challenges in access to standardised weighing scales and capacities to calculate BMI.^51 53^

Findings mainly from prospective cohort studies show that lifestyle factors, such as lack of physical activity, low fruit and vegetable consumption, alcohol consumption and tobacco use, are linked to pre-diabetes and T2D.^54^,^56^ The current study, however, did not find significant associations, possibly related to self-reported biases and the cross-sectional design of the WHS+. Future studies with longitudinal designs could better capture the long-term impacts of lifestyle factors on T2D risk. Another well-known risk factor is the family history of diabetes. As this variable was unfortunately not collected, we were not able to include it in our analysis.

### Implications of the study

While the factors associated with pre-diabetes and UDD have been extensively investigated internationally, this study represents the first national representative analysis within the Cambodian context. As a nation undergoing rapid economic and lifestyle changes, Cambodia’s experience provides a valuable model for understanding and addressing the growing diabetes epidemic in transitioning low-income and middle-income countries worldwide. The identification of key risk factors enables the development of more precise strategies for the prevention and early detection of T2D. The strong, independent associations of elevated BMI and waist circumference with UDD underscore their immediate clinical utility. As non-invasive, cost-effective indicators, they are ideal for initial risk stratification in primary care and community settings. This allows healthcare systems to prioritise individuals for more intensive follow-up and monitoring, as recommended by international guidelines (ADA, the US Preventive Services Task Force).^51 57^

Early detection and intervention of pre-diabetes through lifestyle changes or medication can reduce the risk of progression to diabetes by up to 49% and prevent more severe T2D.^58 59^ This study highlights the importance of enhanced prevention and T2D screening, especially for higher-risk groups. Successful lifestyle interventions have been seen in various countries, including the USA, Finland, Japan, India and China.^60^,^63^ Although this study did not demonstrate significant associations, promoting healthy foods and more stringent regulation of unhealthy products would be expected to show (indirect) effects in the long term.^64^,^69^

### Strengths and limitations

A key strength of the WHS+Cambodia is its large, nationally representative sample size and the use of HbA1c for diagnosing pre-diabetes and T2D, which provides a reliable assessment of the glycaemic status. Limitations include the cross-sectional design, which limits causal inference and potential reporting biases in self-reported lifestyle factors. Additionally, while HbA1c is a widely accepted diagnostic tool, variations in cut-off values across studies may affect prevalence comparisons. Future research should consider longitudinal approaches to understand risk trajectories and intervention effectiveness better.

## Conclusions

This study demonstrated a high prevalence of pre-diabetes and UDD in Cambodia, underscoring the critical need for targeted public health intervention. Significant risk factors included urban residence, advanced age, overweight and obesity, increased waist circumference, elevated blood pressure and high triglyceride levels. Prevention and screening efforts should prioritise these high-risk populations. A comprehensive multisectoral approach encompassing healthcare services, community-based initiatives and health promotion strategies is imperative to mitigate these risks and enhance diabetes management in Cambodia.

## Supplementary material

10.1136/bmjopen-2025-102715 online supplemental file 1

10.1136/bmjopen-2025-102715 online supplemental file 2

10.1136/bmjopen-2025-102715 online supplemental figure 1

10.1136/bmjopen-2025-102715 online supplemental table 1

10.1136/bmjopen-2025-102715 online supplemental table 2

## Data Availability

Data are available on reasonable request.
