# Supplementary material for: Prevalence and factors associated with pre-diabetes and undiagnosed diabetes in Cambodia: cross-sectional study based on the World Health Survey Plus 2023
Source: BMJ Open. 2026 Jan 14;16(1):e102715. doi: 10.1136/bmjopen-2025-102715 (PMC12815103; doi:10.1136/bmjopen-2025-102715)
Supplement: online supplemental file 1 [file bmjopen-16-1-s001.pdf]

RESEARCH PROTOCOL

WORLD HEALTH SURVEY PLUS

Principal Investigator

Prof. Sopheab HENG,

Deputy Director of National Institute of Public Health

Co-Principal Investigator

Prof. Chhorvann CHHEA

Director of National Institute of Public Health

Dr. Nawi NG

Professor, University of Gothenburg, Sweden

Coordinator

Mr. Srean CHHIM

Senior Researcher, National Institute of Public Health

12 July 2022

INTRODUCTION

The overwhelming and crucial importance of achieving Universal Health Coverage (UHC), and other Health Related Sustainable Development Goals (HR-SDGs) in health policy discussions within the global health community is driving a proliferation of new estimates to track progress from increasingly complex analytical models. However, considerable limitations and uncertainty about quality remain regarding the primary input data on which these modelled estimates are based. This uncertainty extends to data to measure and evaluate many of the HR-SDGs. The UN agencies, such as World Health Organization (WHO), United Nations Children's Fund (UNICEF), The United Nations Population Fund (UNFPA) and the World Bank, institutions like the Institute for Health Metrics and Evaluation and Measure, and various donor agencies are contributing to the increasing volume and frequency of new data generation and estimates. These global health estimates are much needed and valuable tools in monitoring trends, predicting future health challenges, and guiding interventions. However, while considerable advances have been made, the efforts to generate results that will help to monitor progress towards the HR-SDGs (which go beyond SDG 3[1]) and UHC also obscure a rather critical weakness in the analytical foundations: namely, there is sparse empirical data that can be used as the basis for the sophisticated modelling required – or to inform national policymaking. Additionally, the WHS+ will collect data on the determinants of health outcomes, including social determinants and household economic status, in order to provide the appropriate explanations for the differences in the health outcomes. The WHS+ will also collect data on subjective well-being to inform the relationship between health and well-being. While many existing surveys, such as the World Bank's Living Standards and Measurement Surveys, might collect some of this information in a piecemeal manner, the WHS+ will collect this data in an omnibus surveys to examine the relationships within the household and individuals who form the sample to provide insights that are otherwise unavailable.

The proliferation of health-related estimates is also driven in part by the growing number of organizations with their own programmatic goals, indicators and targets, including WHO's 13th General Programme of

Work (GPW13) Impact Framework. This framework includes the triple billion targets, one billion more people benefitting from universal health coverage, one billion more people better protected from health emergencies, and one billion more people enjoying better health and well-being by 2023, and 46 indicators for progress towards achieving the HR-SDGs, with a key focus on achieving UHC. These GPW13 indicators are aligned with the SDGs and UHC indicators but, nonetheless, require WHO member states to develop data reporting mechanisms. WHO recognizes that monitoring the multitude of indicators across many international agreements and national priorities will require robust data generation systems. WHO's GPW13, therefore, places a strong focus on the importance of open data systems, with emphasis on country-led and -owned data collection.

One proven method to improve population health estimates is through strengthening routine and periodic data generation efforts and national analytical capacity. WHO's recent SCORE assessment (https://www.who.int/data/stories/score-global-report-2020--a-visual-summary) has clearly demonstrated that a large number of low- and middle-income countries still lack strong data and health information systems to monitor the impact of interventions in a timely and effective manner. Strengthening these systems takes time and sustained attention.

One element of health systems strengthening includes implementation of multi-topic health surveys that provide robust and high-quality data for monitoring health trends and determinants. Household health surveys are a reliable method of addressing many data gaps and form a critical component of national data and health information systems. For instance, 80 of the 232 SDG indicators come from household surveys, according to the Inter-Secretariat Working Group on Household Surveys.[2] These include 29 of the 59 health-related SDG indicators. The option of using any additional surveys to assess progress towards SDGs should, ideally, be integrated into a nationally-led, regular and coordinated system and schedule of household health surveys.

While most countries have some form of household health surveys as part of their data and health information system, especially in low resource settings these are heavily donor dependent and not nationally owned and institutionalized.

WHO's World Health Survey Plus (WHS+) is proposed as the next generation of an integrated multi-topic, multi-mode household health survey for all member states learning from the experience of conducting household health surveys over the last two decades.

The **WHS+** protocol and implementation plan builds on experiences from the 2003 World Health Survey (WHS) and longitudinal WHO Study on global AGEing and adult health (SAGE), which have provided national and sub-national level data for a number of countries. The WHS and SAGE studies were reviewed by WHO's ethical review committee as well as national institutional review boards. The data from these surveys still remain invaluable today in providing internationally comparable information in large datasets not available in other omnibus household health surveys. The foundations of the **WHS+** will also aim to build on and work with other large survey programmes, including the World Bank's Living Standards Measurement Study (LSMS) or Household Income and Expenditure Survey (HIES), USAID's Demographic and Health Surveys (DHS), UNICEF's Multiple Indicator Cluster Survey (MICS), and WHO's World Mental Health Survey.

AIMS AND OBJECTIVES

The aim of the **WHS+** is to generate valid, reliable, comparable and timely information on a range of health and well-being outcomes and determinants of public health importance in nationally representative target populations. The results from the **WHS+** will support WHO member states to track progress on country-specific objectives and towards UHC and HR-SDG targets, and WHO's Impact Framework approved by Member States.

Primary Objectives

1) Create a robust multi-functional data collection platform for generating data to monitor progress on national priorities, HR-SDGs and UHC
2) Support countries to generate, analyse and use high-quality data to fill data gaps
3) Develop standardized, computerized data collection strategies that enable speedier data collection and more reliable comparison across countries.

Additional Objectives

1) Develop capacities in using evidence for policy
2) Institutionalize a regular data generation platform in countries
3) Provide tools and methods for better sequencing and coordination between studies
4) Pursue improvements in integrating survey data into health information systems
5) Provide methods for the inclusion of neglected topics and population groups
6) Build on post-census surveys with up-to-date sampling frames
7) Introduce a suite of data collection and analysis tools to use alongside National Statistics Offices and the United Nations Statistics Division

METHODOLOGY

Sampling and sample size

The standard sampling design for the **WHS+** will be a nationally representative stratified multi-stage cluster sampling of households when traditional area-based sampling frames derived from a recent population census or large-scale household sample survey are available.

The sample size will be approximately 6000 households including a margin to support an expected non-response of up to 20 percent. A larger sample size will be considered if sub-national estimates are required.

The typical design will stratify the areas in the frame by regions that are specified as core domains of interest. For each of these regions, further area stratification may or may not take place, depending on the quality of the available frame information.

Sampling of areas defined as primary sampling units (PSUs) will be carried out using probability proportional to size (PPS). Typical size measures are numbers of households or residents from the most recent population census or some other similar source.

The number of PSUs to be selected in each stratum are obtained by dividing corresponding stratum sample sizes by the expected number of households an interviewer can cover during the time allocated for completing data collection work within a sampled PSU.

Sub-sampling of households in each sampled PSU will be carried out after updating the list of households in the PSU.

The **WHS+** will explore an alternate sampling frame approach – gridded population datasets – to improve and extend representativeness of the results. Especially where up-to-date sampling frames are unavailable, this approach helps to establish robust, representative study samples.

The sampling frame is defined based on micro-level population estimates. Satellite imagery and its derived products can be used to construct a population layer, using an easy, workable method that divides the area of interest into grids. The sampling design reflects the characteristics of the random field by

combining contextual stratification and is proportional to population size sampling. The parameters of the grid samples include coverage, frame, design, strata, target and sample size[2] .

The **WHS+** team will work with in-country colleagues to determine the most appropriate way of identifying the target population and then advise on how best to identify, select and contact the sample. Through this innovative and tailored approach, the **WHS+** teams will be able to collect detailed, granular information about different population groups.

The units of analysis will be the household and individual. The target population is adults, 18 years and older, residing within individual households, institutionalized population excluded. Inclusion criteria are age 18 years and older (unless countries want younger populations included and can obtain ethical approvals and appropriate procedures for the **WHS+** implementation) and ability to provide informed consent.

Prospective household surveys will use interview modes that best suit the country situation. The **WHS+** will provide options to collect data in a number of different ways, taking advantage of the latest technologies for face-to-face computer assisted personal interviews (CAPI), computer-assisted telephone interviews (CATI) and online surveys. Translation and back-translation protocols, as per WHO's instrument translation guidelines, will be used to ensure a variety of main languages are available for the interviews in each country.

The type of data to be collected includes interview and biomarker data. The data collection period is open-ended, starting in 2022 for baseline surveys. A 5-year survey cycle will be pursued for repeat cross-sectional design surveys.

---

[2] Thomson et al Int. J Health Geogr (2020)19:34 https://doi.org/10.1186/s12942-020-00230-4

The household member who is most knowledgeable about the household will be invited to complete the household questionnaire as the household informant. The interviewers will be trained to identify the household member through a set of probing questions that will be clarified during the training and in the interviewer manual for the WHS+. This person will then be interviewed following informed consent. Eligible household members will be listed in the household roster and will be randomly selected by the CAPI or by the CATI programme, including approaches such as a random-digit dial, to complete the individual interview. Typically one eligible respondent will be selected to complete the individual questionnaire per household. When the module on reproductive and child health is included in the individual questionnaire, a second respondent, a woman of reproductive age, will be randomly selected to complete this module only if the initial eligible respondent is not a woman of reproductive age. The individual respondent will be an adult 18+ years in age and who gives consent to be interviewed.

When a country wants to implement a longitudinal cohort study the **WHS+** design will take this into account and the sample will be designed accordingly. In countries where there has been previous implementation of a longitudinal study like SAGE attempts will be made to follow up the previous sample and account for attrition.

Data Collection

Standardized survey content, set of methods, interviewer training, and translation protocols will be used.

The survey content will be selected in close collaboration with countries from multiple modules developed for the **WHS+** survey instrument, which have been guided by HR-SDG, UHC and WHO Impact Framework indicators, as well as by the goal of filling data gaps in assessing health, determinants, and wellbeing.

A suite of survey modules that meet international standards, include reliable and valid interview questions, appropriate measurement approaches, and that incorporates the latest health and biological tests has been developed, with direct input from technical experts within and outside WHO. Short and long versions of the individual modules will also be made available depending on technical needs and expert advice. Many of the questions have been harmonized with validated instruments, building on work by groups such as the International Household Survey Network (http://www.ihsn.org/health-modules), but where specific cross-cultural elements arise, questions may need to be tested in cognitive interviews and pilot studies before the main surveys start.

The suite of survey module options for the household and individual questionnaires (including core and expanded versions of these modules and special topic modules) would help to further tailor topics within the survey instruments in order to meet specific country needs. The approach in each country will be unique – but will mirror the approaches likely to be used in all participating countries: stand-alone (core or expanded); stand-alone in parallel with other multi-country survey programmes such as the Demographic and Health Surveys and the Multiple Indicator Cluster Surveys; and/or, nested within other large studies as core or special modules. In the latter scenario a probabilistic sample will be selected from the larger study to supplement data missing from the larger study and to then link data from both studies. There will be no duplication of information collected and will be a synergistic exercise.

Member states will generate a household questionnaire and individual questionnaire from a set of standardized modules covering a wide range of topics (see example table below). A suite of modules covering a wide array of priority health issues could be used to create the **WHS+** instrument as a stand-alone survey.

Table 1. Example of the suite of standardized survey modules covering a range of thematic areas that countries could select to create their WHS+ instrument

Household	Individual	Special
Introduction and consent	Introduction and consent	
Recontact information	Sociodemographic characteristics	Community surveys, Structural determinants of health
Household roster including health insurance coverage and immunization schedules	Health state (health status and functioning) and disability	Disability survey
Housing (including water and sanitation and use of clean fuels)	Anthropometric measurements, performance tests (vision, hearing, gait speed, grip strength, cognition), blood tests	Mortality – WHO Verbal Autopsy Questionnaires, birth history/ orphanhood, sibling survivorship
Household and family support networks and transfers	Health risks (tobacco, diet, physical activity, alcohol, social determinants of health, food insecurity, salt intake)	Violence against women, violence against children
Assets and household income	Chronic conditions and level of health care provision for those conditions	Mental health survey
Household consumption expenditure (including health expenditure)	Infectious diseases and health care coverage (incl. antimicrobial resistance)	
Deaths in the household	Reproductive, maternal and child health	Prevention of unsafe abortion
	Health care utilization (including health system responsiveness and satisfaction)	Facility surveys
	Subjective Well Being	WHO Quality of Life surveys or happiness and wellbeing surveys

A number of objective measures and tests will also be available. Anthropometric measurement options include (height, weight, waist and hip circumferences). Measured tests include blood pressure measurement and a blood sample via finger prick. Approximately 70 microliters of blood will be collected from a finger prick and will be used immediately in point-of-care devices to assess anaemia, blood sugar levels and cholesterol levels. The test will use new, disposable sterile instruments that are clean and completely safe. None of the samples will be stored for further analyses. The results will be shared with the respondent on the same day – and with information about whether the respondent might want to visit their local health care provider. All materials used to collect the sample – including any remnant blood from the finger prick and from the test – will be destroyed as part of standard universal precaution practices (at a health facility or laboratory). Standard infection prevention and control procedures as per WHO guidelines and national procedures will be followed in terms of using procedures such as handwashing, use of gloves, sterilization of puncture sites for collection of capillary blood, and use of appropriate disinfectants. The lancets used for the collection of capillary blood will be collected in a sharps container and then disposed of as per national protocols (e.g., by autoclaving). The other materials will be sealed in a plastic bag and disposed of as per national guidelines (e.g., by incinerating). No venous blood samples will be collected for subsequent analysis in laboratories.

Performance tests include near and distant vision, hearing screen, a timed 4-m walk, grip strength, and cognition.

As an example, a stand-alone **WHS+** household questionnaire could include (1) a household roster and modules about the dwelling, income, transfers in and out of the household, assets and expenditures; (2) an individual questionnaire with modules on health and its determinants, disability, chronic conditions and risk factors, subjective well-being, health care utilization and health systems responsiveness; and, (3) a verbal autopsy module questionnaire to ascertain the probable cause of death for deaths in the household in the 24 months prior to interview.

However the **WHS+** need not always mean creating an additional household survey: modules could be integrated into other ongoing economic and health surveys (for example, a selection of key modules could be included within a planned Demographic and Health Survey).

In another example, integrating the **WHS+** into a DHS in an African country, for instance, may choose to include core (short) versions of modules on health, chronic conditions, health care utilization, and health care expenditures.

Interview process

In each country, the in-country **WHS+** lead and team will organize translation and interview team training and testing before interviews start. Face-to-face interviews will be conducted, after obtaining informed consent, using computer-assistance (CAPI) in all countries. Laptop computers or netbooks will be required for the in-person interviews. Interview duration of 30-45 minutes for the household questionnaire and 60 minutes for the individual questionnaire will be targeted. Future rounds of data collection, including interim rapid assessments, will use the **WHS+** sampling frame but could be implemented through mobile phone or online surveys.

Training for the technical/IT aspects of the CAPI will be provided online via training videos – as well as in person over one week prior to study implementation. Similarly training for anthropometrics and biomarkers collection will be online and in person and be provided by technical experts. Content training in each country with survey teams will take place in close collaboration with the WHO country office and WHO HQ teams. WHO will provide ongoing support throughout the data collection period.

Study setting and data collection period

The **WHS+** would be aligned with the 2020 census round and run as a stand-alone endeavour or be integrated into an existing data collection platform as mentioned above.

We plan to work with an initial set of 10 countries in 2022/2023, to work through the methodologies and reporting mechanisms. The longer-term goal is to implement the **WHS+** in approximately 50 countries. In Cambodia, the number of provinces selected in this survey will depend on the sampling using PPS. It is likely that all provinces will be included.

It is expected that data collection will take 12 weeks. Data will be submitted regularly to WHO - with regular feedback and communication between the respective country survey lead investigator/team, and the WHO country office and WHO Geneva. Analyses and publication of results will be conducted by WHO in partnership with the member state. The raw data collected in the study and the methods developed to collect and analyse the raw data belong jointly and severally to WHO and the provider.

Brief summary of potential analyses

A table of the most recent household and population estimates by stratifiers (locality(urban/rural), region/state/province, sex, age-groups) will be required prior to starting interviews. Household weights for analysis at household level and individual weights for analysis at person level will be calculated. These weights will be based on the selection probability at each stage of sampling.

Household weights will be post-stratified by region/state/province and locality. Individual weights will be post-stratified by region/state/province, sex and age-groups (18-29, 30-39, 40-49, 50-59, 60-69, 70+). Post stratification adjustments will be based on the most recent household and population estimates provided by the National Statistics Office.

Weighted social and demographic characteristics of respondents will be summarized using descriptive statistics.

Further topic-specific analyses will be led by topical experts and adjusted for the complex survey design. Each module will include guidance on how to compute key indicators linked to the UN SDGs or WHO Impact Framework, for example. Additional multi-variable modelling to examine individual-level and country-level variables, or sub-group analyses, associated with primary or secondary outcomes will be organized by the interested analyst. Analytical scripts will be generated and shared by WHO HQ to ensure results are reproducible – and to speed up the dissemination of results.

Quality assurance

WHO HQ will support in-country teams, who will be responsible for quality assurance mechanisms throughout training and data capture. Collected data will be stored on password-protected secure servers.

Quality control measures will be employed with frequent data transfers to WHO. Detailed non-response specifics will be required. Survey team supervisors will be monitoring missing data/outliers and will conduct random checks and retests in specified sample populations. **WHS+** teams implementing the study in each member state will be expected to maintain regular contact with WHO, providing updates and troubleshooting where necessary.

Dissemination plan

Results will be disseminated in technical reports, scientific papers and through monitoring and evaluation mechanisms for the HR-SDGs and WHO Impact Framework. Participating countries will be encouraged to develop policy briefs and to communicate research findings to relevant policy makers. Individual results from the point-of-care-testing will be provided to the respondents at the time of data collection and they will be asked to contact their health care provider as needed. The high level summary results from the survey will also be communicated through a short document in the local language to the community leaders and sent to the research participants.

The **WHS+** micro-data that will be made publicly available will be deidentified, with provisions to ensure data cannot be linked to individual respondents for analysts interested in cross-country comparisons.

Ethical review processes

WHO's Ethical Review Committee will continue to review and assess the study, design and outcomes. Each member state will have the responsibility of engaging with their respective ethical review bodies and obtaining approval. Informed consent is required for participation in the study.

Safety considerations

WHS+ will present no greater than minimal risk to respondents. Some countries will choose to include survey modules that contain several questions that may be sensitive in some local settings, including questions for women respondents about sexual behaviour, abortion, and violence. The respondent will be allowed to stop the survey at any point and leave out questions that they do not wish to answer.

WHS+ will remove all personal identifiers from resulting datasets. During data collection, accessibility to data sets will be controlled by in-country **WHS+** leads, who will shape final decisions about the final public-use dataset. Data sharing agreements will be signed between participating country institutions and WHO HQ.

For those with blood screening tests or physical measurements that suggest needed attention by a qualified health service provider, survey teams will encourage a respondent to engage with their regular health service provider, or a local provider.

In light of the ongoing COVID-19 pandemic, additional safety measures will be developed and enacted to ensure survey teams, interviewers and respondents do not become vectors for further virus spread. The training workshops for data collectors will strictly adhere to country COVID-19 protocols. If required,

and if in-person training will not be possible, the entire training will be organised online. Implementing agencies will only recruit fully vaccinated people for the field team. Entire field teams will have to agree upon and follow detailed COVID-19 prevention protocol.

Data collectors will be provided with personal protective equipment (PPE) kits during the data collection process. PPE kits include: surgical masks, non-surgical masks, gloves, goggles, face shields, gowns and N95 masks. Supervisors and research officers will ensure that the data collectors are wearing PPE kits during data collection.

Compensation

Compensation for participation in the interview process with be left to the discretion of the implementing agency in consultation with the local research ethics review committee.

Resources and funding

Initial funding will come through WHO HQ. Resource mobilization efforts are under way to support the implementation of the **WHS+**. As a collaboration, all participating member states will be expected to contribute resources to **WHS+** implementation, analyses and dissemination of results.
