# Supplementary material for: Prevalence and factors associated with pre-diabetes and undiagnosed diabetes in Cambodia: cross-sectional study based on the World Health Survey Plus 2023
Source: BMJ Open. 2026 Jan 14;16(1):e102715. doi: 10.1136/bmjopen-2025-102715 (PMC12815103; doi:10.1136/bmjopen-2025-102715)
Supplement: online supplemental figure 1 [file bmjopen-16-1-s003.pdf]

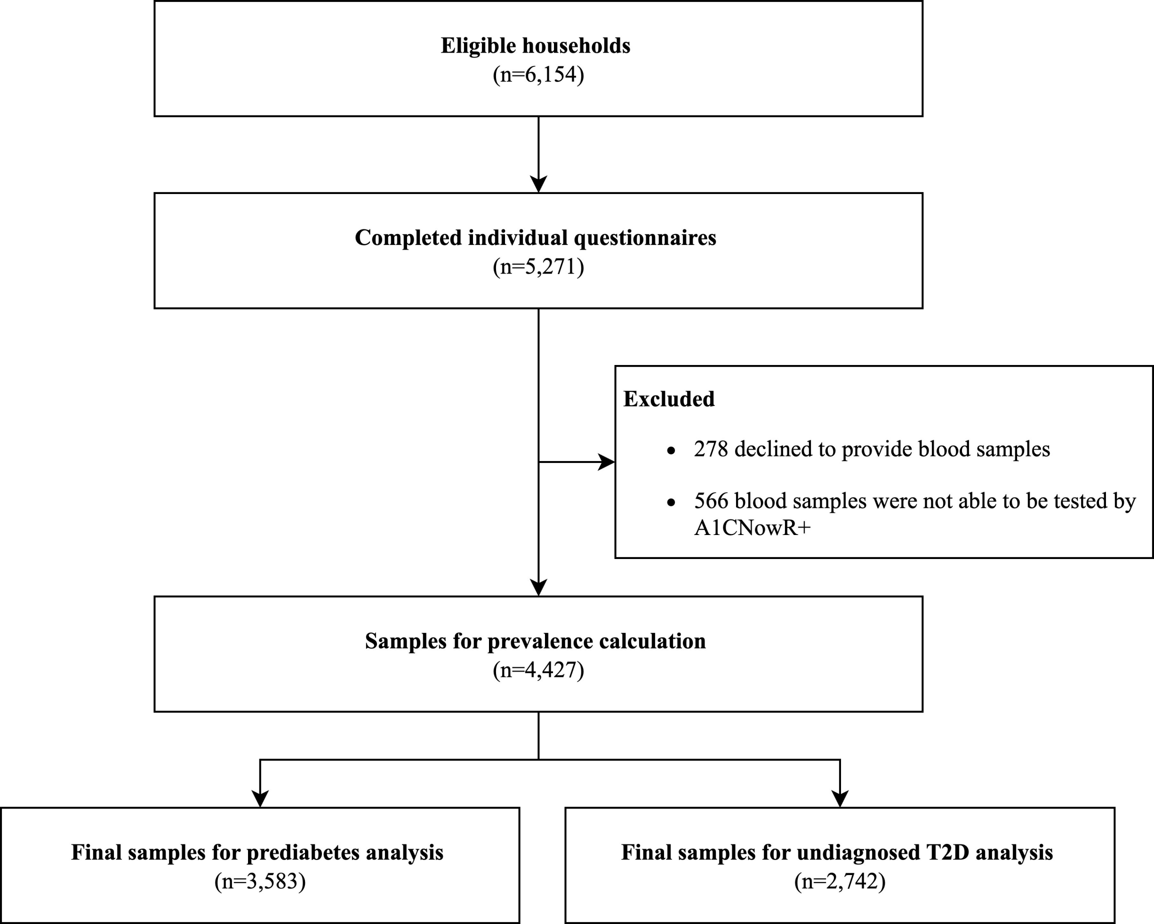
Eligible households
(n=6,154)
Completed individual questionnaires
(n=5,271)
Excluded
278 declined to provide blood samples
566 blood samples were not able to be tested by A1CNowR+
Samples for prevalence calculation
(n=4,427)
Final samples for prediabetes analysis
(n=3,583)
Final samples for undiagnosed T2D analysis
(n=2,742)
