# Supplementary material for: Prevalence and factors associated with pre-diabetes and undiagnosed diabetes in Cambodia: cross-sectional study based on the World Health Survey Plus 2023
Source: BMJ Open. 2026 Jan 14;16(1):e102715. doi: 10.1136/bmjopen-2025-102715 (PMC12815103; doi:10.1136/bmjopen-2025-102715)
Supplement: online supplemental table 1 [file bmjopen-16-1-s004.docx]

Supplementary Table 1. Bivariate analysis of factors associated with prediabetes and UDD

|  | **Prediabetes** | | | **Undiagnosed T2D** | | |
| --- | --- | --- | --- | --- | --- | --- |
| **Characteristic** | **No Diabetes**,  N = 2,379 | **Prediabetes**,  N = 1,204 | **p-value** | **No Diabetes**,  N = 2,379 | **Diagnosed T2D**,  N = 363 | **p-value** |
| Type of community |  |  | **<0.001** |  |  | **<0.001** |
| Rural | 1,589 (72.4) | 726 (27.6) |  | 1,589 (93.4) | 178 (6.6) |  |
| Urban | 790 (62.3) | 478 (37.7) |  | 790 (83.2) | 185 (16.8) |  |
| Sex of participant |  |  | **<0.001** |  |  | 0.3 |
| Male | 711 (64.1) | 395 (35.9) |  | 711 (90.8) | 89 (9.2) |  |
| Female | 1,668 (72.8) | 809 (27.2) |  | 1,668 (88.6) | 274 (11.4) |  |
| Age group (years) |  |  | **<0.001** |  |  | **<0.001** |
| 18-29 | 443 (81.7) | 101 (18.3) |  | 443 (99.3) | 3 (0.7) |  |
| 30-39 | 619 (69.0) | 207 (31.0) |  | 619 (94.3) | 18 (5.7) |  |
| 40-49 | 508 (62.1) | 261 (37.9) |  | 508 (88.2) | 54 (11.8) |  |
| 50+ | 809 (54.6) | 635 (45.4) |  | 809 (71.5) | 288 (28.5) |  |
| MET category |  |  | 0.3 |  |  | 0.4 |
| Active (>=600) | 1,641 (69.6) | 825 (30.4) |  | 1,641 (89.0) | 236 (11.0) |  |
| Inactive (<600) | 734 (66.6) | 378 (33.4) |  | 734 (90.7) | 127 (9.3) |  |
| Fruit and vegetable consumption |  |  | 0.2 |  |  | 0.7 |
| <5 | 1,362 (70.0) | 666 (30.0) |  | 1,362 (89.9) | 216 (10.1) |  |
| >=5 (adequate) | 1,017 (66.7) | 538 (33.3) |  | 1,017 (89.2) | 147 (10.8) |  |
| Smoking |  |  | **0.024** |  |  | >0.9 |
| Non | 1,788 (70.3) | 846 (29.7) |  | 1,788 (89.6) | 271 (10.4) |  |
| Smoker | 591 (63.5) | 358 (36.5) |  | 591 (89.6) | 92 (10.4) |  |
| Alcohol disorder category |  |  | 0.8 |  |  | 0.9 |
| Low risk | 1,880 (68.4) | 965 (31.6) |  | 1,880 (89.5) | 304 (10.5) |  |
| High risk | 499 (69.2) | 239 (30.8) |  | 499 (89.9) | 59 (10.1) |  |
| BMI category |  |  | **<0.001** |  |  | **<0.001** |
| Underweight (<18.5) | 207 (77.4) | 94 (22.6) |  | 207 (97.9) | 8 (2.1) |  |
| Normal (18.5-24.9) | 1,505 (75.2) | 586 (24.8) |  | 1,505 (92.5) | 171 (7.5) |  |
| Overweight (25.0-29.9) | 553 (53.9) | 414 (46.1) |  | 553 (86.5) | 132 (13.5) |  |
| Obese (>=30.0) | 103 (49.8) | 102 (50.2) |  | 103 (67.7) | 34 (32.3) |  |
| Waist circumference category (cm) |  |  | **0.006** |  |  | **<0.001** |
| High | 226 (57.2) | 207 (42.8) |  | 226 (80.2) | 104 (19.8) |  |
| Normal | 2,149 (69.8) | 993 (30.2) |  | 2,149 (91.0) | 253 (9.0) |  |
| Total Cholesterol |  |  | **0.029** |  |  | **0.004** |
| Normal | 1,976 (69.5) | 949 (30.5) |  | 1,976 (91.7) | 244 (8.3) |  |
| Elevated (≥ 240mg/dL) | 267 (60.1) | 199 (39.9) |  | 267 (83.0) | 81 (17.0) |  |
| Total Triglycerides |  |  | **<0.001** |  |  | **<0.001** |
| Normal | 1,332 (75.4) | 525 (24.6) |  | 1,332 (95.3) | 86 (4.7) |  |
| Elevated (≥ 150mg/dL) | 946 (60.9) | 632 (39.1) |  | 946 (85.5) | 233 (14.5) |  |
| Having hypertension | 637 (58.2) | 471 (41.8) | **<0.001** | 637 (71.8) | 257 (28.2) | **<0.001** |
