# Supplementary material for: Prevalence and factors associated with pre-diabetes and undiagnosed diabetes in Cambodia: cross-sectional study based on the World Health Survey Plus 2023
Source: BMJ Open. 2026 Jan 14;16(1):e102715. doi: 10.1136/bmjopen-2025-102715 (PMC12815103; doi:10.1136/bmjopen-2025-102715)
Supplement: online supplemental table 2 [file bmjopen-16-1-s005.docx]

Supplementary Table 2. Multivariable logistic regression using unimputed data using case-available datasets

|  | **Prediabetes** | | | **Undiagnosed T2D** | | |
| --- | --- | --- | --- | --- | --- | --- |
| **Characteristic** | **aOR** | **95% CI** | **p-value** | **aOR** | **95% CI** | **p-value** |
| Type of community |  |  |  |  |  |  |
| Rural | — | — |  | — | — |  |
| Urban | 1.2 | 1.0, 1.4 | **0.047** | 1.4 | 1.1, 1.8 | **0.002** |
| Sex of participant |  |  |  |  |  |  |
| Female | — | — |  | — | — |  |
| Male | 2.0 | 1.2, 3.3 | **0.005** | 2.8 | 1.1, 6.9 | **0.027** |
| Age group (years) |  |  |  |  |  |  |
| 18-29 | — | — |  | — | — |  |
| 30-39 | 1.4 | 1.0, 2.1 | **0.038** | 1.9 | 1.0, 3.9 | 0.060 |
| 40-49 | 2.3 | 1.6, 3.3 | **<0.001** | 3.6 | 2.0, 7.3 | **<0.001** |
| 50+ | 3.6 | 2.6, 5.1 | **<0.001** | 5.9 | 3.2, 12 | **<0.001** |
| MET category (cut-off at 600 minutes/week |  |  |  |  |  |  |
| Active (>=600) | — | — |  | — | — |  |
| Inactive (<600) | 1.1 | 0.9, 1.2 | 0.549 | 1.1 | 0.9, 1.4 | 0.515 |
| Former and current smoker |  |  |  |  |  |  |
| Non | — | — |  | — | — |  |
| Smoker | 1.1 | 0.9, 1.3 | 0.373 | 1.0 | 0.7, 1.3 | 0.850 |
| Alcohol disorder category |  |  |  |  |  |  |
| Low risk | — | — |  | — | — |  |
| High risk | 0.9 | 0.8, 1.1 | 0.440 | 1.2 | 0.9, 1.6 | 0.226 |
| BMI category |  |  |  |  |  |  |
| Normal (18.5-24.9) | — | — |  | 1.8 | 1.0, 3.5 | 0.050 |
| Underweight (<18.5) | 1.2 | 0.9, 1.6 | 0.253 | — | — |  |
| Overweight (25.0-29.9) | 1.6 | 1.4, 2.0 | **<0.001** | 2.6 | 1.4, 5.1 | **0.003** |
| Obese (>=30.0) | 2.0 | 1.4, 2.8 | **<0.001** | 4.5 | 2.2, 9.5 | **<0.001** |
| Waist circumference category (cm) |  |  |  |  |  |  |
| Normal | — | — |  | — | — |  |
| High | 1.3 | 1.0, 1.7 | 0.061 | 2.1 | 1.5, 2.9 | **<0.001** |
| Total Cholesterol |  |  |  |  |  |  |
| Normal | — | — |  | — | — |  |
| Elevated (≥ 240mg/dL) | 1.2 | 0.9, 1.5 | 0.183 | 1.2 | 0.9, 1.7 | 0.138 |
| Total Triglycerides |  |  |  |  |  |  |
| Normal | — | — |  | — | — |  |
| Elevated (≥ 150mg/dL) | 1.2 | 1.1, 1.5 | **0.007** | 1.6 | 1.3, 2.1 | **<0.001** |
| Having hypertension |  |  |  |  |  |  |
| No | — | — |  | — | — |  |
| Yes | 1.1 | 0.9, 1.3 | 0.482 | 1.4 | 1.1, 1.8 | **0.003** |
| Sex of participant * Age group (years) |  |  |  |  |  |  |
| Male * 30-39 | 0.8 | 0.4, 1.4 | 0.432 | 0.4 | 0.1, 1.1 | 0.072 |
| Male * 40-49 | 0.6 | 0.3, 1.0 | 0.066 | 0.3 | 0.1, 0.8 | **0.013** |
| Male * 50+ | 0.5 | 0.3, 0.8 | **0.006** | 0.4 | 0.1, 1.0 | **0.044** |
| Abbreviations: CI = Confidence Interval, aOR = Ajusted Odds Ratio | | | | | | |
