# Supplementary material for: Prevalence and factors associated with pre-diabetes and undiagnosed diabetes in Cambodia: cross-sectional study based on the World Health Survey Plus 2023
Source: BMJ Open. 2026 Jan 14;16(1):e102715. doi: 10.1136/bmjopen-2025-102715 (PMC12815103; doi:10.1136/bmjopen-2025-102715)
Supplement: Supplementary file 6 [file bmjopen-2025-102715.reviewer_comments.pdf]

PEER REVIEW HISTORY

BMJ Open publishes all reviews undertaken for accepted manuscripts. Reviewers are asked to complete a checklist review form and are provided with free text boxes to elaborate on their assessment. These free text comments are reproduced below.

ARTICLE DETAILS

Title (Provisional)

Prevalence and factors associated with prediabetes and undiagnosed diabetes in Cambodia: Cross-sectional study based on the World Health Survey Plus 2023

Authors

Chhim, Srean; Ku, Grace Marie V.; Kowal, Paul; Te, Vannarath; Rithisey, Mony; Ngor, Chamnab; Walton, Poppy; Maung, Khin Thiri; Ng, Nawi; Por, Ir; Klipstein-Grobusch, Kerstin; Chhea, Chhorvann; Sopheab, Heng

---

VERSION 1 - REVIEW

**Reviewer**	**1**
**Name**	**Dewi, Ayu Putu Madri**
**Affiliation**	**Amsterdam UMC Location AMC, Epidemiology and Data Science**
**Date**	**16-Apr-2025**
**COI**	**None**

Manuscript Title: Prevalence and factors associated with prediabetes and undiagnosed diabetes in Cambodia: Cross-sectional study based on the World Health Survey Plus 2023

Dear authors,

I am an Open Science researcher, who has checked your manuscript for its adherence to a number of Open Science practices.

Below you find the checklist that I used to assess your manuscript for adherence to a number of these practices.

Based on this assessment, I would suggest the following refinements/additions:

1. Availability of a protocol: Please provide a (link to the) the protocol.

2. Pre-registration: Please provide information on whether and where the study was pre-registered.

3. Open Materials: OK.

4. Open Data: Given the confidentiality of the data, please specify whether de-identified, metadata, or a subset of the data can still be made publicly available.

Note, if this paper is accepted, you will be required to add a data availability statement and may be required to share data by BMJ/BMJ Open Policy. For more information, please see: https://authors.bmj.com/policies/data-sharing/ ; https://bmjopen.bmj.com/pages/authors

5. Open Code: Please provide (a link to) the R code used for the analyses in this study.

Note that for papers accepted at The BMJ you will be required to provide a supplementary file with the code if this paper is accepted. For more information, please see Data sharing section on the website: https://authors.bmj.com/policies/data-sharing/ (The BMJ); https://bmjopen.bmj.com/pages/authors (BMJ Open)

6. Citations: Please provide citations for your data/codes.

7. Software and tools used are described: OK

8. Open access publication: OK

9. Reporting checklist provided: OK

10. Preprint: Please state explicitly whether and where the preprint of this study can be found.

**Reviewer**	**2**
**Name**	**Abera, Rodas Getachew**
**Affiliation**	**Addis Ababa University**
**Date**	**23-May-2025**
**COI**	**None**

Thank you for allowing me to review the manuscript. The study findings provided important information on understanding the current state of pre-type 2 diabetes and undiagnosed T2D in Cambodia. They also presented the associated factors and highlighted improvement strategies.

1. Abstract, Methods Section: The abstract should be self-explanatory. It lacks important information, such as how participants were selected (sampling technique), the study period, multivariate analysis, and the statistical significance (P-value =0.005) used.

2. Data Collection: The specific date of the data collection period should be mentioned. In addition to the A1CNowR+ point-of-care device having a built-in quality control function, what other measures have you taken to confirm the reliability of the result? Have you cross-checked it with the central laboratory measurement of HbA1c, as the number of samples tested is too many for a point-of-care device?

3. Page 16, Prevalence of pre-T2D and undiagnosed diabetes: The comma in the stated number is misplaced (41,04) and needs correction.

4. What is the point of using a 5,271 sample size if 844 participants were excluded? Only 4,104 were used to calculate the prevalence and determine the associated factors. Why not remove 5,271 from the manuscript and stick with the final 4,104 participants?

**Reviewer**	**3**
**Name**	**Mashili, Fredirick**
**Affiliation**	**Muhimbili University of Health and Allied Sciences, Physiology**
**Date**	**03-Jul-2025**
**COI**	**None**

Focusing on pre diabetes and undiagnosed diabetes is a unique attribute and the greatest strength of this study. With the increasing burden of diabetes and its complications, screening and early detection are commendable. This study partly addresses early detection of diabetes by investigating prevalence and factors associated with an earlier diabetes stage (pre diabetes). Despite its novelty, addressing the following questions and comments will add value and strengthen the manuscript

GENERAL COMMENTS

1. Beyond prevalence, the manuscript adds very little to the existing body of evidence. All the factors found to be associated with diabetes and pre diabetes are widely known. This means that the discussion part of the manuscript should try as much to justify why further highlighting those factors is important. Further discussion on the implication of results to policy, public health and practice will improve the quality of the manuscript.

2. The authors have used HbA1C to define pre diabetes. While it is OK, there is still a lot of discussion around results based on fasting glucose, 2hours glucose (OGTT), and HbA1C with OGTT being superior compared to the rest. The authors should somehow discuss this, and justify why they chose HbA1C over others.

SPECIFIC COMMENTS AND QUESTIONS

1. Family history of diabetes is a known determinant of pre DM and DM. I wonder why the authors did not include it, or even discuss it.

2. I am not sure if pre T2DM is a correct term to use. Please countercheck that

3. Why are the authors combining pre DM and DM when discussing the associated factors. While the two might have common factors, undiagnosed DM is mainly due to social cultural

and social economic than biological factors. It really confuses combining the two. I feel like the two should be treated separately. Undiagnosed diabetes present an access challenge where as pre diabetes mainly present a biological challenge resulting from other factors.

4. Which physical activity questionnaire did the authors used. From their description of what was analyzed, looks like active commuting was not considered in calculating the metabolic equivalent of task. This could be a reason of having very low levels of physical activity in a population predominantly from rural areas. Please clarify the questionnaire used and add more details on how METS were derived and calculated. Additionally, the categorization of METS into intensity doesn't seem to follow WHO criteria. Please mention which criteria you used. Also try to discuss the rural, urban differences in both risk factors, pre DM and DM.

VERSION 1 - AUTHOR RESPONSE

**Reviewer: 1**<br>**Ms. Ayu Putu Madri Dewi, Amsterdam UMC Location AMC**<br>**Comments to the Author:**<br>**Manuscript Title: Prevalence and factors associated with prediabetes and undiagnosed diabetes in Cambodia: Cross-sectional study based on the World Health Survey Plus 2023**	
Dear authors,<br>I am an Open Science researcher, who has checked your manuscript for its adherence to a number of Open Science practices. Below you find the checklist that I used to assess your manuscript for adherence to a number of these practices.<br><br>Based on this assessment, I would suggest the following refinements/additions:	We are grateful to the reviewer for your thorough assessment of our manuscript's adherence to Open Science practices. We have addressed each point as detailed below.
1. Availability of a protocol: Please provide a (link to the) the protocol.	In response to this request, we have now included the study protocol as a supplementary file. We indicated this in text at the end of the paragraph under the Data source section that:<br><br>*"Original protocol is available in the Supplementary File 1."*
2. Pre-registration: Please provide information on whether and where the study was pre-registered.	We confirm that this study, being a secondary analysis of a cross-sectional survey and not a clinical trial, was not pre-registered.
3. Open Materials: OK.	The relevant materials were provided

4. Open Data: Given the confidentiality of the data, please specify whether de-identified, metadata, or a subset of the data can still be made publicly available. Note, if this paper is accepted, you will be required to add a data availability statement and may be required to share data by BMJ/BMJ Open Policy. For more information, please see: https://authors.bmj.com/policies/data-sharing/ ; https://bmjopen.bmj.com/pages/authors	We thank the reviewer for this important point. The de-identified dataset used for this analysis is publicly available from the World Health Organization at the following link. We have included a formal data availability statement in the manuscript under the section "DATA AVAILABILITY STATEMENT" to reflect this.<br><br>*"Data are available upon reasonable request through https://apps.who.int/healthinfo/systems/surveydata/index.php/collections/central"*
5. Open Code: Please provide (a link to) the R code used for the analyses in this study.<br>Note that for papers accepted at The BMJ you will be required to provide a supplementary file with the code if this paper is accepted. For more information, please see Data sharing section on the website: https://authors.bmj.com/policies/data-sharing/ (The BMJ); https://bmjopen.bmj.com/pages/authors (BMJ Open)	As requested, the R code used for all analyses presented in this study has been provided as a supplementary file [Supplementary file 2].<br><br>In the manuscript, at the end of the Data Analysis section, we added:<br><br>*"R codes are provided as Supplementary File 2."*
6. Citations: Please provide citations for your data/codes.	The citation specially for this data is not available, but the link to the original dataset of WHO was proved in the Data availability Statement section.
7. Software and tools used are described: OK	We thank the reviewer for noting this. To provide further clarity, we have specified in the Methods section that the analysis was conducted using R programming, highlighting the use of key packages such as tidyverse, MASS, gtsummary, and MICE.
8. Open access publication: OK	Thanks for noticing.
9. Reporting checklist provided: OK	Thanks for noticing.

10. Preprint: Please state explicitly whether and where the preprint of this study can be found.	The pre-print version was available online via link https://www.medrxiv.org/content/10.1101/2025.03.09.25322832v2
**Reviewer: 2**<br>**Mr. Rodas Getachew Abera, Addis Ababa University**	
Comments to the Author: Thank you for allowing me to review the manuscript. The study findings provided important information on understanding the current state of pre-type 2 diabetes and undiagnosed T2D in Cambodia. They also presented the associated factors and highlighted improvement strategies.	We thank the reviewer for your constructive feedback and efforts in reviewing our manuscript. We have carefully revised the manuscript based on these comments, which are addressed point-by-point below.
1. Abstract, Methods Section: The abstract should be self-explanatory. It lacks important information, such as how participants were selected (sampling technique), the study period, multivariate analysis, and the statistical significance (P-value =0.005) used.	We thank the reviewer for these helpful suggestions to improve the clarity of the abstract. We have revised the abstract to include: (a) the sampling technique used for participant selection, (b) the specific study period, (c) a reference to the multivariate logistic regression analysis, and (d) the statistical significance level ($p < 0.05$). The abstract is now formatted according to the journal's guidelines and is more self-explanatory.
2. Data Collection: The specific date of the data collection period should be mentioned. In addition to the A1CNowR+ point-of-care device having a built-in quality control function, what other measures have you taken to confirm the reliability of the result? Have you cross-checked it with the central laboratory measurement of HbA1c, as the number of samples tested is too many for a point-of-care device?	Thank you for these important questions. We have addressed them as follows:<br><br>▪ **Data Collection Period:** We have specified the exact data collection period. The first sentence of the 'Data Collection' section now reads:<br><br>*Data were collected between March 12 and May 31, 2023."*<br><br>▪ **Device Reliability**: While we did not perform a direct cross-check with a central laboratory for this specific study, we have expanded the Methods section to discuss the device's validation and quality control features.<br><br>*"The device features a built-in quality control function to ensure reliable results. It automatically detects issues such as insufficient or excessive blood volume, abnormal haemoglobin density, and extreme temperatures. This device is designed for research purposes or as a diagnostic tool when laboratory-based*

	HbA1c is not available. A systematic review and meta-analysis analysed biases using A1cNow, reported a low average bias ranging from –0.70% (95% confidence interval, CI –0.82 to 0.58%) to +0.67% (95% CI 0.52–0.82%), compared to laboratory-based testing.<br>*During the survey, we also measured lipids and random blood glucose levels using a CardioChek PA Analyzer (PTS Diagnostics Headquarters, Whitestown, IN USA) 30 and blood pressure using an Automatic Blood Pressure Monitor HEM-7120 (OMRON Healthcare, Kyoto, Japan).”*
3. Page 16, Prevalence of pre-T2D and undiagnosed diabetes: The comma in the stated number is misplaced (41,04) and needs correction.	We thank the reviewer for highlighting these points. We have corrected the typographical error in the number. We also agree that presenting the initial sample size was confusing. We have revised the manuscript to state the final analytical sample size of 4,427 participants from the outset, after accounting for the 844 exclusions. The text now flows more logically, focusing only on the participants included in the final analysis (n=4,427) throughout the manuscript.
4. What is the point of using a 5,271 sample size if 844 participants were excluded? Only 4,104 were used to calculate the prevalence and determine the associated factors. Why not remove 5,271 from the manuscript and stick with the final 4,104 participants?	As stated in the response to comment 3 above, we revised the manuscript highlighting the n=4,427 participants on whom analyses are based.
**Reviewer: 3**<br>**Dr. Fredirick Mashili, Muhimbili University of Health and Allied Sciences**	
Focusing on pre diabetes and undiagnosed diabetes is a unique attribute and the greatest strength of this study. With the increasing burden of diabetes and its complications, screening and early detection are commendable. This study partly addresses early detection of diabetes by investigating prevalence and factors associated with an earlier diabetes stage (pre diabetes). Despite its novelty, addressing the following questions and	We are grateful for the reviewer’s thoughtful comments and advice, which have helped us significantly improve the manuscript. We carefully addressed your comments point-by-point as below.

comments will add value and strengthen the manuscript	
GENERAL COMMENTS<br>1. Beyond prevalence, the manuscript adds very little to the existing body of evidence. All the factors found to be associated with diabetes and pre diabetes are widely known. This means that the discussion part of the manuscript should try as much to justify why further highlighting those factors is important. Further discussion on the implication of results to policy, public health and practice will improve the quality of the manuscript.	We thank the reviewer for this insightful comment. While we acknowledge that the identified risk factors are well-established globally, the investigation within the Cambodian context is novel and provides crucial local evidence. To better highlight the manuscript's contribution, we have revised the Discussion section to focus on the public health and policy implications of these findings specifically for Cambodia. We now elaborate on how this evidence can inform targeted national screening programs, prevention strategies, and health system resource allocation in the country.<br><br>*"*<br>*While the factors associated with prediabetes and UDD have been extensively investigated internationally, this study represents the first such analysis within the Cambodian context. The identification of key risk factors enables the development of more precise strategies for the prevention and early detection of T2D."*
2. The authors have used HbA1C to define pre diabetes. While it is OK, there is still a lot of discussion around results based on fasting glucose, 2hours glucose (OGTT), and HbA1C with OGTT being superior compared to the rest. The authors should somehow discuss this, and justify why they chose HbA1C over others.	This is an important methodological point. We have added a paragraph to the Discussion section addressing the choice of HbA1c. We acknowledge that the Oral Glucose Tolerance Test (OGTT) is considered the gold standard. However, we justify the use of HbA1c based on its practicality and feasibility in a large-scale population-based survey setting, as it does not require fasting. We also now explicitly discuss the potential for underestimation of prevalence when using HbA1c alone and have added this as a limitation of the study.<br><br>The second paragraph of the discussion started with *"This study used HbA1c to estimate prediabetes prevalence in Cambodia. Comparing our prevalence to the global prevalence presents challenges owing to variations in diagnostic tools and cutoff criteria. While there is no consensus on prediabetes screening strategies, studies using HbA1c have reported a higher prevalence than those using FBG, but a lower prevalence than OGTT, which is considered the superior method."*
SPECIFIC COMMENTS AND QUESTIONS<br>1. Family history of diabetes is a known determinant of pre DM and DM. I wonder	We agree with the reviewer that family history of diabetes is a key risk factor. Unfortunately, data on this variable were not collected as part of the original World Health Survey Plus. We have now explicitly acknowledged this as a significant limitation in the 'Strengths and limitations of

why the authors did not include it, or even discuss it.	this study' section and in the Discussion at end of the paragraph 6.<br><br>"Another well-known risk factor is the family history of diabetes. As this variable was unfortunately not collected, we were not able to include it in our analysis."
2. I am not sure if pre T2DM is a correct term to use. Please countercheck that.	Pre-T2D or pre-T2DM can be used interchangeably (https://www.health.harvard.edu/diseases-and-conditions/type-2-diabetes-mellitus-a-to-z). We appreciate the reviewer's comment on terminology. After reviewing guidelines from major organizations such as the WHO and the American Diabetes Association, we agree that 'prediabetes' is the more standard and widely accepted term. We have replaced all instances of 'pre-T2D' with 'prediabetes' throughout the manuscript for clarity and consistency.
3. Why are the authors combining pre DM and DM when discussing the associated factors. While the two might have common factors, undiagnosed DM is mainly due to social cultural and social economic than biological factors. It really confuses combining the two. I feel like the two should be treated separately. Undiagnosed diabetes present an access challenge where as pre diabetes mainly present a biological challenge resulting from other factors.	The reviewer raises a critical point. Our rationale for combining prediabetes and undiagnosed diabetes was to identify common risk factors for general dysglycemia compared to the normoglycemic reference group, which is useful for primary prevention strategies. However, we recognize the reviewer's point that factors leading to a lack of diagnosis (e.g., social, economic, healthcare access) may differ from purely biological risk factors for prediabetes. To address this, we have now added a paragraph in the Discussion to explore this nuance. We have also clarified our analytical approach and its justification in the Methods section.
4. Which physical activity questionnaire did the authors used. From their description of what was analyzed, looks like active commuting was not considered in calculating the metabolic equivalent of task. This could be a reason of having very low levels of physical activity in a population predominantly from rural areas. Please clarify the questionnaire used and add more details on how METS were derived and calculated. Additionally,	We are very grateful to the reviewer for this important observation. Upon re-examination, we confirmed that commuting activities were indeed inadvertently omitted from our initial MET calculation. We have now corrected this by recalculating the total weekly MET minutes using the complete Global Physical Activity Questionnaire (GPAQ) data, including work, non-work, and commuting activities. This has resulted in a more accurate reflection of physical activity levels, with the proportion of inactive participants decreasing from 53% to 35.2%. All relevant tables and text in the Results section have been updated accordingly. We have also added a more detailed description of the GPAQ and MET calculation to the Methods section, as suggested.

the categorization of METS into intensity doesn't seem to follow WHO criteria. Please mention which criteria you used. Also try to discuss the rural, urban differences in both risk factors, pre DM and DM.	In description of this variable in Table 1, we have some key information:<br><br>"Weekly physical activity was assessed ***by use of the Global Physical Activity Questionnaire (GPAQ) and converted to Metabolic Equivalents of Task (MET) minutes***. Participants self-reported the frequency and duration of moderate- and vigorous-intensity physical activity at work, non-work, and **commuting**. MET values were assigned (4 for moderate and 8 for vigorous), and the total weekly MET minutes were calculated. Participants were then classified **into two groups: < 600 MET-minutes, and >=600** (recommended).

VERSION 2 - REVIEW

Reviewer 2

Name Abera, Rodas Getachew

Affiliation Addis Ababa University

Date 05-Sep-2025

COI

Thank you for addressing the comments and providing satisfactory explanations. The manuscript has been improved greatly. Wish you good luck!!

Reviewer 3

Name Mashili, Fredirick

Affiliation Muhimbili University of Health and Allied Sciences, Physiology

Date 06-Sep-2025

COI

The authors have addressed all my previously raised concerns. I am particularly impressed by the way the physical activity analysis has been expanded to include ACTIVE commuting which was initially omitted.

To further strengthen the scientific implication of the manuscript and place the findings into the global context the authors should consider adding the following;

1. Discuss in short the PH implication of the associations of BMI and WC with undiagnosed DM. These are biological/clinical and not social economic factors and the authors should discuss why knowing them is important (follow up and monitoring for those with high BMI and WC, or rather considering a more accurate test like OGTT among those with higher BMI and WC to avoid missing the diagnosis).

2. Where the authors have discussed policy implications of their findings for Cambodia, they should add why is this important globally.

VERSION 2 - AUTHOR RESPONSE

<table>
<tr><th>Comments</th><th>Responses</th></tr>
<tr><td colspan="2">Reviewer: 3
Dr. Fredirick Mashili, Muhimbili University of Health and Allied Sciences</td></tr>
<tr><td>To further strengthen the scientific implication of the manuscript and place the findings into the global context the authors should consider adding the following;

1. Discuss in short the PH implication of the associations of BMI and WC with undiagnosed DM. These are biological/clinical and not social economic factors and the authors should discuss why knowing them is important (follow up and monitoring for those with high BMI and WC, or rather considering a more accurate test like OGTT among those with higher BMI and WC to avoid missing the diagnosis).</td><td>We thank the reviewer for this insightful comment. Crucially, as the reviewer noted, a high BMI or WC should not be an endpoint but a trigger for further action. The most critical implication is the targeted use of more definitive diagnostic tests, such as the oral glucose tolerance test (OGTT) or HbA1c, in these high-risk individuals. This two-step approach, screening with anthropometric measures followed by confirmatory testing, is an efficient strategy to reduce the number of missed diagnoses and enable earlier intervention, ultimately improving long-term health outcomes.

We have expanded the discussion to clarify the public health implications of our findings:

"The strong, independent associations of elevated BMI and waist circumference with undiagnosed type 2 diabetes (T2D) underscore their immediate clinical utility. As non-invasive, cost-effective indicators, they are ideal for initial risk stratification in primary care and community settings. This allows healthcare systems to prioritize individuals for more intensive follow-up and monitoring, as recommended by international guidelines (ADA, USPSTF) [61, 67]."</td></tr>
<tr><td>2. Where the authors have discussed policy implications of their findings for Cambodia,</td><td>We thank the reviewer for this suggestion. We have added a sentence to clarify the global importance:</td></tr>
</table>

they should add why is this important globally.	*"While the factors associated with prediabetes and UDD have been extensively investigated internationally, this study represents the first national representative analysis within the Cambodian context. As a nation undergoing rapid economic and lifestyle changes, Cambodia's experience provides a valuable model for understanding and addressing the growing diabetes epidemic in transitioning low- and middle-income countries worldwide."*
